# Transcriptome profiling identifies ABA mediated regulatory changes towards storage filling in developing seeds of castor bean (*Ricinus communis* L.)

**DOI:** 10.1186/2045-3701-4-33

**Published:** 2014-06-30

**Authors:** Umashankar Chandrasekaran, Wei Xu, Aizhong Liu

**Affiliations:** 1 Key Laboratory of Tropical Plant Resource and Sustainable Use, Xishuangbanna Tropical Botanical Garden, Chinese Academy of Sciences, 88 Xuefu Road, Kunming 650223, China; 2 Kunming Institute of Botany, Chinese Academy of Sciences, 132 Lanhei Road, Kunming 650201, China; 3 University of Chinese Academy of Sciences, Beijing 100049, China

**Keywords:** ABA signaling, Castor bean, Developing seeds, High throughput RNA-Seq, Storage reserve, ELISA

## Abstract

**Background:**

The potential biodiesel plant castor bean (*Ricinus communis*) has been in the limelight for bioenergy research due to the availability of its genome which raises the bar for genome-wide studies claiming advances that impact the “genome-phenome challenge”. Here we report the application of phytohormone ABA as an exogenous factor for the improvement of storage reserve accumulation with a focus on the complex interaction of pathways associated with seed filling.

**Results:**

After the application of exogenous ABA treatments, we measured an increased ABA levels in the developing seeds cultured *in vitro* using the ELISA technique and quantified the content of major biomolecules (including total lipids, sugars and protein) in treated seeds. Exogenous ABA (10 μM) enhanced the accumulation of soluble sugar content (6.3%) followed by deposition of total lipid content (4.9 %). To elucidate the possible ABA signal transduction pathways towards overall seed filling, we studied the differential gene expression analysis using Illumina RNA-Sequencing technology, resulting in 2568 (1507-up/1061-down regulated) differentially expressed genes were identified. These genes were involved in sugar metabolism (such as glucose-6-phosphate, fructose 1,6 bis-phosphate, glycerol-3-phosphate, pyruvate kinase), lipid biosynthesis (such as ACS, ACBP, GPAT2, GPAT3, FAD2, FAD3, SAD1 and DGAT1), storage proteins synthesis (such as SGP1, zinc finger protein, RING H2 protein, nodulin 55 and cytochrome P450), and ABA biosynthesis (such as NCED1, NCED3 and beta carotene). Further, we confirmed the validation of RNA-Sequencing data by *Semi-quantitative* RT-PCR analysis.

**Conclusions:**

Taken together, metabolite measurements supported by genes and pathway expression results indicated in this study provide new insights to understand the ABA signaling mechanism towards seed storage filling and also contribute useful information for facilitating oilseed crop functional genomics on an aim for utilizing castor bean agricultural and bioenergy use.

## Background

The plant hormone abscisic acid (ABA) is broadly involved in various stress-related responses and developmental regulation in higher plants [1]. Many studies have demonstrated that ABA participates in regulating organ development such as flower, fruit, root and seed in diverse plants [2-4]. In particular, ABA regulates several important aspects of seed development such as embryogenesis [5], endosperm development [6] and the biosynthesis of storage reserves (lipids, proteins and starch) during seed filling [7]. Endogenous ABA levels are closely associated to storage reserve accumulation in developing seeds [8,9]. The pattern of endogenous ABA accumulation in developing seeds appeared to be varied in different plants; however, most cases observed showed that endogenous ABA accumulation was tightly associated with seed growth and storage material accumulation in developing seeds [4,8]. On the other hand, exogenous ABA application enhanced the levels of TAG’s (nine fold increase) in somatic embryos of *Picea glauca* and rapeseed cultures [7,10]. In addition, application of exogenous ABA increased the levels of total sugar content as well as reducing sugars in cultured seedling of *Phaseolus vulgaris* and in cell cultures of oilseed rape [11,12]. A 19 kDa oil body protein encoding oleosin and a desaturase lipid gene were found to be up regulated, correlating to the physiological changes after exogenous ABA treatment in *B. napus* embryo cultures [7]. In *Arabidopsis*, ABA responsive genes LEC2 (CCAAT family), FUS3, ABI3 and a maize VP1 (*viviparous 1*) were identified to be critical for the embryogenesis [13]. In maize, the ABA responsive gene *VP8* was demonstrated to have an essential role in coordinating embryo and endosperm development [13]. Besides, ABI3 and FUS3 genes were found to be critical in mediating the accumulation of storage materials in *Arabidopsis* seeds [13,14]. More than forty ABA response genes involved in embryogenesis, endosperm development and the accumulation of storage materials were identified in developing seeds of rice [15]. Although diverse ABA responsive genes involved in seed development and storage material accumulation have been identified in different plants, the molecular basis for ABA dependent physiological responses in developing seeds is far from understanding.

Investigating the transcriptional changes of responsive genes to ABA signal by applying exogenous ABA as stimulant is of great help to identify critical ABA regulators in developing seeds. Many such efforts have been made and identified diverse genes involved in many aspects of seed development in different plants [7]. In particular, high-throughout sequencing technology has recently become a powerful tool that allows to profile a genomic expression pattern and to measure modest changes among different samples at unprecedented perspectives [16,17]. However limited information is known about the holistic transcriptional changes of responsive genes to ABA signals in developing seeds.

Castor bean (*Ricinus communis*, Euphorbiaceae) is one of the most important non-edible oilseed crops. Its seed is a highly specialized storage organ for accumulating lipids, proteins, and carbohydrates in endosperm [18]. Its seed oils comprised of the unusual hydroxy-fatty acid (i.e. ricinoleic acid) is broadly used in industry and is considered as an ideal and unique feedstock for biodiesel production [19]. Its seed storage protein ricin is extremely toxic and able to be reportedly used as a biochemical weapon or a specific immunotoxin for therapeutic purposes in different cancer treatments [20,21]. Due to economical importance, castor bean has been widely accepted as an agricultural solution for all subtropical and tropical regions that addresses the need for commercial crops with low impute costs and at the same time provides traditional farming with a viable income from current non- productive lands. With increasing demand for production of castor bean seed oils in many countries, breeding and improvement of varieties are drawing great attention from breeders. Further efforts should be made to elucidate the molecular mechanism underlying the regulation of growth and development [22]. Study on the molecular basis of ABA physiological response in developing seeds is of significance for understanding the mechanism controlling seed development and crop improvement in castor bean.

In this study, we inspected the effect of exogenous ABA signaling on the storage material accumulation and preformed high-throughput sequencing to identify the ABA responsive genes in castor bean seed filling. Results obtained here provide new insights into the understanding of molecular mechanisms underlying ABA regulation towards seed development and storage material accumulation in castor developing seeds.

## Results

### Effects of exogenous ABA on seed development

Based on our previous study, the endogenous ABA levels were closely associated with storage material accumulation in developing castor seed tissues [9]. To further examine the effect of ABA signal on storage material accumulation in developing seeds, we applied exogenous ABA as a stimulant in a nutrient medium to culture developing castor seeds *in vitro*. According to our previous observation the endogenous ABA accumulation of developing castor seeds at ca. 21 day after pollinated (DAP) was low, and associated with the initiation of rapid storage reserve accumulation occurring at this stage [9,23]. Thus the developing castor seeds at ca. 21 DAP were used for further assay. Initially, four different concentrations (1, 10, 50 and 100 μM) of free form ABA (ethanol dissolved) in media were pre-tested. Seeds cultured with the exogenous ABA at 50, 100 μM concentrations resulted in an abnormal development possibly because of high osmotic potential after 96 h treatment (data not shown). This observation suggested that high ABA concentrations (such as 50 or 100 μM) in MS media cause a negative effect on cultured seed development, consistent with previous observations in rapeseed cultures and *Lesquerella fendleri* cultures [7,24]. Seeds cultured in media with the exogenous ABA at 1 μM concentration did not show any difference in storage reserve accumulation compared to the control (data not shown), whereas seeds cultured in media with the exogenous ABA at 10 μM concentration apparently resulted in an increased accumulation in lipid contents after 96 h treatment. As shown in Table 1, exogenous ABA at 10 μM enhanced the storage material accumulation in cultured seeds after 96 h treatment. The dry weight increased 1.5 mg after ABA treatment. The deposition of total lipid and soluble sugar significantly increased 4.9% (from 1.78 to 3.23 mg^−1^seed, p < 0.05) and 6.3% (from 12.58 to 15.01 mg^−1^seed, p < 0.05), respectively, comparing to the control levels. However, the amount of protein content deposited slightly decreased compared to the control (from 5.9 to 5.2 mg^−1^seed, p < 0.05) (Table 1). Similar effect of exogenous ABA on enhancing lipid deposition preceded by a decrease in protein content has been previously reported in MD embryos of *Brassica napus* L [7].

**Table 1:** Effect of exogenous ABA on seed reserve parameters

**Seed treatment**	**Protein content (mg/seed)**	**Lipid content (mg/seed)**	**Sugar content (mg/seed)**	**Dry Weight (mg/seed)**
**Control**	5.9 ± 1.3	1.78 ± 0.4	12.58 ± 2.5	25.9 ± 1.2
**ABA**	5.2 ± 1.1	3.23 ± 0.8^*^	15.01 ± 2.8^*^	27.4 ± 1.6

^*^Statistically significant at P < 0.05 as student’s t test procedure.

In addition, compartmentalization of lipid classes (neutral lipid-NL) mediated by exogenous ABA (10 μM) based on the TLC plate analysis was explored (Additional file 1: Figure S1). Triricinolein (TR3), the most prominent component in castor bean, is the high polar triglyceride. Triglycerides containing two ricinoleates and one unhydroxylated acyl moiety are less polar (TR2). Triglycerides of two or three unhydroxylated acyl moieties (TR1) are least polar and are found close to the solvent front. TR3 castor lipid class was found to be highly responsive by ABA treatments compared to the control samples followed by TR2 the second major lipid class of castor lipids was also responsive but not as high as that of TR3 comparing to the control sample density (Additional file 1: Figure S1). Minimal increase was observed in TR1 the least triglyceride density compared to control spot percent (Additional file 1: Figure S1). There were no any visual differences in seed morphology observed after ABA treatments during the course of the experiments. To compare the prolific changes caused by ABA signaling towards storage reserves at this stage, lipid content, sugar content and protein content were also measured in developing seeds at different developmental stages (7-63 DAP). From our results, protein synthesis was significantly high during the early-mid seed filling stages (7-35 DAP; 0.3-27.7 mg^−1^seed) comparing to lipid deposition which increased rapidly from 21-35 DAP (0.95-30 mg^−1^seed) although minimal amounts detected during early stages of seed filling (7-14 DAP) (Additional file 2: Figure S2). Synthesis of lipid and protein components declined on a slow rate during the maturation stages (42-63 DAP). On the other hand, high amount of sugar levels were noted only during the early stages (7-21 DAP; 0.8-6.7 mg^−1^seed) after which a rapid decline during the mid-developmental as well as the maturation stages was observed (Additional file 2: Figure S2). This analysis of metabolite levels in developing seeds (*in vivo*) is of fundamental importance to understand the changes caused by ABA treatment at 21 DAP (*in vitro* cultured). The advancement of lipid deposition by ABA in developing seeds depends on the percentage of intrusion in to the seed tissues. To make sure the elicitation of exogenous ABA, we measured the ABA content both in culture medium and treated seeds. The culture medium analyses were performed at 3 different time intervals 6, 24, 72 h after treatment. After 6 h of treatment about 79% of free ABA was detected in the medium which was highly stable until 24 h treatments (24%) after which only minimal amounts (1.5%) were detected in 72 h culture medium. Preference was given to the initial stages (24 h) in treated seeds to limit the catabolism of free form ABA in to its metabolites. Relatively, higher content of ABA was noted in seeds treated (24 h) with exogenous ABA than the control samples (Additional file 3: Figure S3).The data presented (means of five replicates) were statistically significant as determined by student’s *t*-test. The significant changes of storage reserves in castor seed filling after exogenous ABA treatment suggested that ABA play a critical role in mediating storage material metabolism in seed filling.

### Global gene expression profile

To identify ABA responsive regulatory factors and reveal the potential molecular basis of ABA effects on storage material accumulation in castor seed filling, high throughput RNA-Seq analysis was performed using cultured seed tissues with ABA at 10 μM concentrations in MS media and without ABA as controls (see Materials and Methods section) under *in vitro* conditions. As shown in Table 2, approximately 4.8 and 4.9 million tags were yielded with 363864 and 382767 distinct tags in control and ABA treated libraries, respectively. After filtering the low quality reads, adaptor and contaminant sequences and the clean tags were 4601620 and 4670041, with 145057 and 161749 distinct clean tags obtained for control and ABA libraries, respectively. Castor bean gene abundance that included 31221 unigenes was used for mapping tags and counting the tag abundance. Among the sequences, genes with CATG site accounted for 90.89%. Finally, 2195481 and 1967523 clean tags were obtained and mapped to genome among which 1914805 and 2344576 clean tags were aligned to a gene obtaining 1849271 and 2310867 unique tags for control and ABA treated libraries, respectively. In order to assess the sequence quality and sequencing depth, the tag coverage and saturation was analyzed for each library. As showed in Table 2, when the sequencing count reached 2 million tags, the number of detected genes tended towards saturation, suggesting that our sequencing depth was sufficient to detect the differential expression of ABA responsive genes in libraries.

**Table 2:** Categorization and summary of abundance of RNA-Seq tags in two libraries (control and ABA treated)

**Summary**	**Control**	**ABA**
Total tags (raw tags)	4825495	4895913
Clean tags	4601620	4670041
Tags mapped to gene	1914805 (41.6%)	2344576 (50.2%)
Unique tags mapped to gene	1849271 (40.1%)	2310867 (49.4%)
Tags mapped to genome	2195481 (47.7%)	1967523 (42.1%)
Unknown tags	491334 (10.6%)	357942 (7.66%)

Clean tags are tags that remained after filtering out dirty tags (low quality tags) from raw data.

### ABA mediates differential gene expression in castor developing seeds

**Figure 1:**
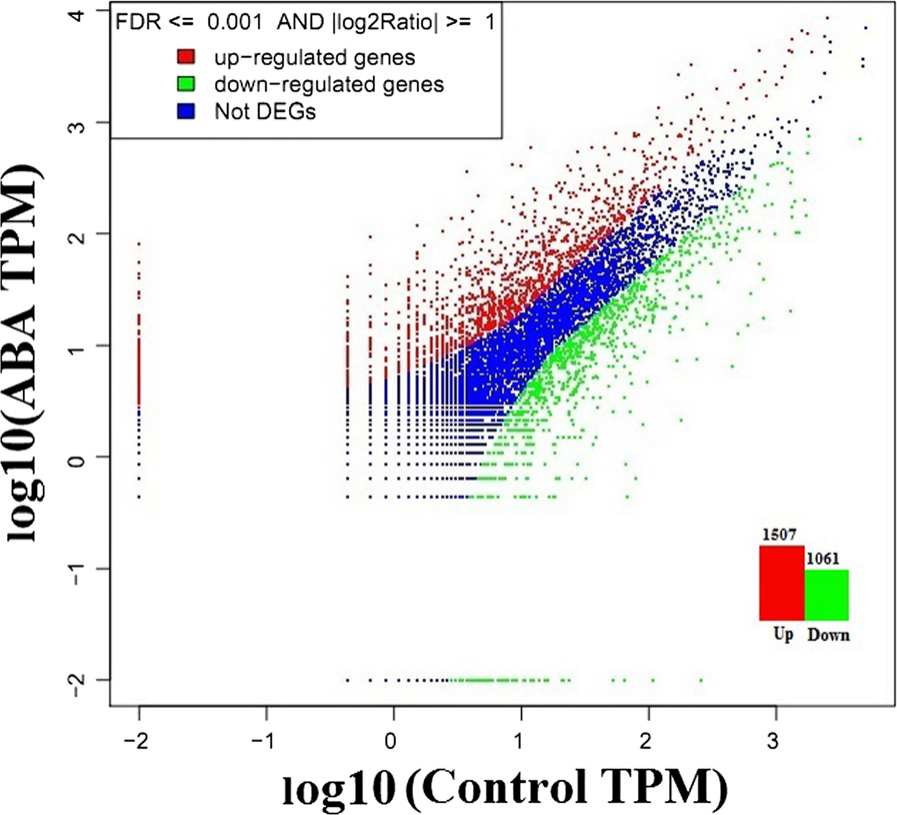
Global analysis of differentially expressed genes in the ABA treatment and its control.

**Figure 2:**
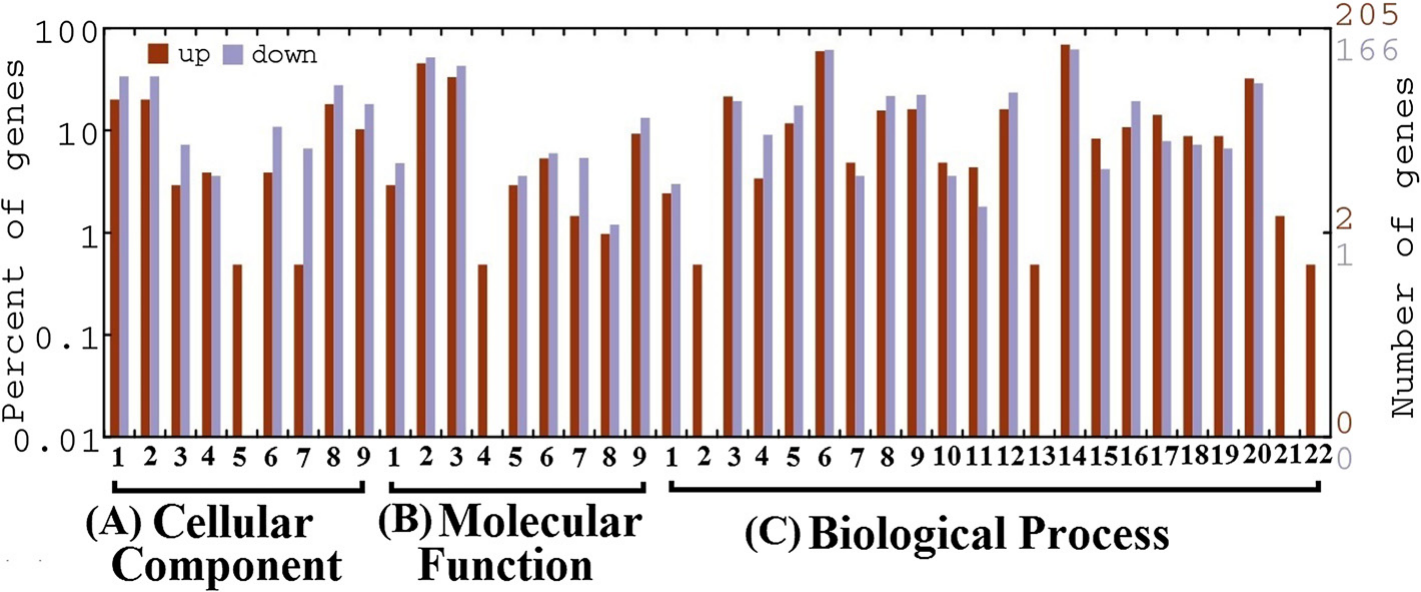
**Gene Ontology functional enrichment analysis of unigenes differentially expressed in control vs ABA treated seeds.** Unigenes were assigned to three categories: **(A)** cellular components: 1-cell, 2-cell part, 3-envelope, 4-extracellular region, 5- extracellular region part, 6-macromolecular complex, 7-membrane enclosed lumen, 8-organelle, 9-organelle part; **(B)** molecular functions: 1-antioxidant, 2-binding, 3-catalytic, 4-electron carrier, 5-enzyme regulator, 6-molecular transducer, 7-structural molecule, 8-translation regulator, 9-transporter; and **(C)** biological processes: 1-anatomical structure formation, 2-biological adhesion, 3-biological regulation, 4-cellualar component biogenesis, 5-cellular component organization, 6-cellular process, 7-death, 8-developmental process, 9-establishment of localization, 10-growth, 11-immune system process, 12-localization, 13-locomotion, 14-metabolic process, 15-multi-organism process, 16-multicellular organism process, 17-pigmentation, 18-reproduction, 19-reproductive process, 20-response to stimulus, 21-rhythmic process, 22-viral reproduction.

**Figure 3:**
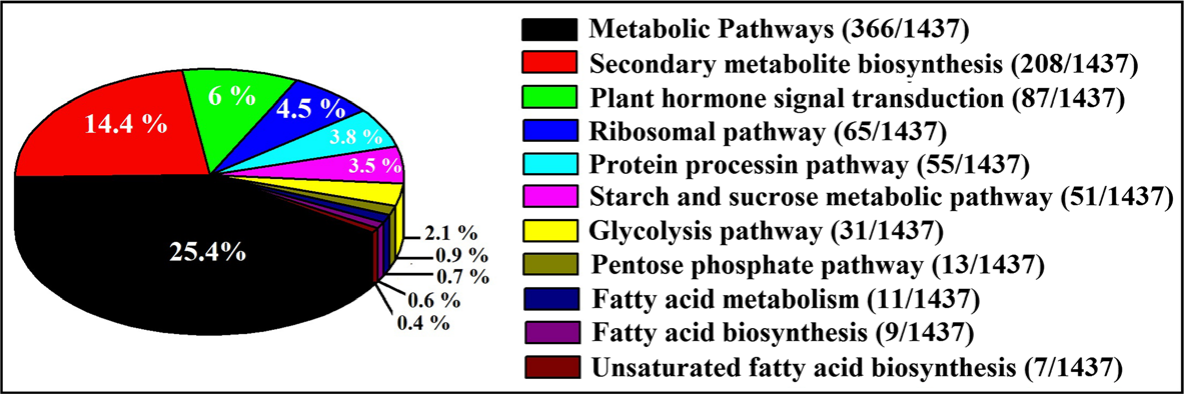
KEGG pathway enrichment analysis for all differentially expressed genes identified from two libraries (control and ABA).

To assess the transcriptional changes in ABA treated seeds; a stringent algorithm method was applied to identify differentially expressed genes from the normalized digital gene expression (DGE) library by comparing the ABA treated samples with control. The results showed that 2568 genes had P <0.05, false discovery rates (FDR) < 0.001, and fold-change ≥ 1 in the comparisons of ABA vs control which were identified as differentially expressed genes (Figure 1). Among these, 1507 genes were significantly up-regulated and 1061 genes were down-regulated in response to ABA treatment (Additional file 4: Table S1). Genes with differential expression responses included a wide variety of regulatory and metabolic processes and were classified into three categories based on their Gene Ontology (GO) terms (Figure 2). In each of the three categories (cellular component, molecular function and biological process) of the GO classification, ‘cell’, ‘cell part’, ‘binding’, ‘catalytic activity’, ‘cellular process’, ‘stimuli response’ and ‘metabolic process’, terms were dominant, respectively (Figure 2). We also noticed a high-percentage of genes from categories of, ‘developmental processes’, ‘cellular component’, ‘biological adhesion’, ‘immune system process’, ‘reproductive process’ and ‘growth’ and only a few genes from terms of ‘extra cellular part’, ‘electron carrier’, ‘locomotion’, and ‘rhythmic process’ (Figure 2). The GO analysis showed that the major functions of the identified genes were involved in various biological processes, suggesting that ABA have comprehensive impacts on seed development at this stage (21 DAP).Genes with similar expression patterns usually imply functional correlation. To evaluate our annotation process, the annotated unigenes were classified based on pathway analysis which can further help our understanding of the biological functions and interactions of genes (Figure 3). A total of 1437 unigenes were differentially expressed with pathway annotations assigned to 122 pathways in the KEGG database. The most represented pathways included “metabolic pathways” (containing 366 genes), “biosynthesis of secondary metabolites pathways” (containing 208 genes), “plant hormone signal transduction” (containing 87 genes), “protein processing” (containing 55 genes) and “starch metabolism” (containing 51 genes). Notably, main pathways were closely linked to changes in glycolysis pathway, sugar metabolism, lipid biosynthesis and hormone signal transduction (Figure 3). These identified genes would provide critical clues to clone and identify key ABA responsive genes which mediate sugar, lipid and protein biosynthesis in developing seeds of castor bean.

### ABA responsive genes associated with storage material metabolisms

**Figure 4:**
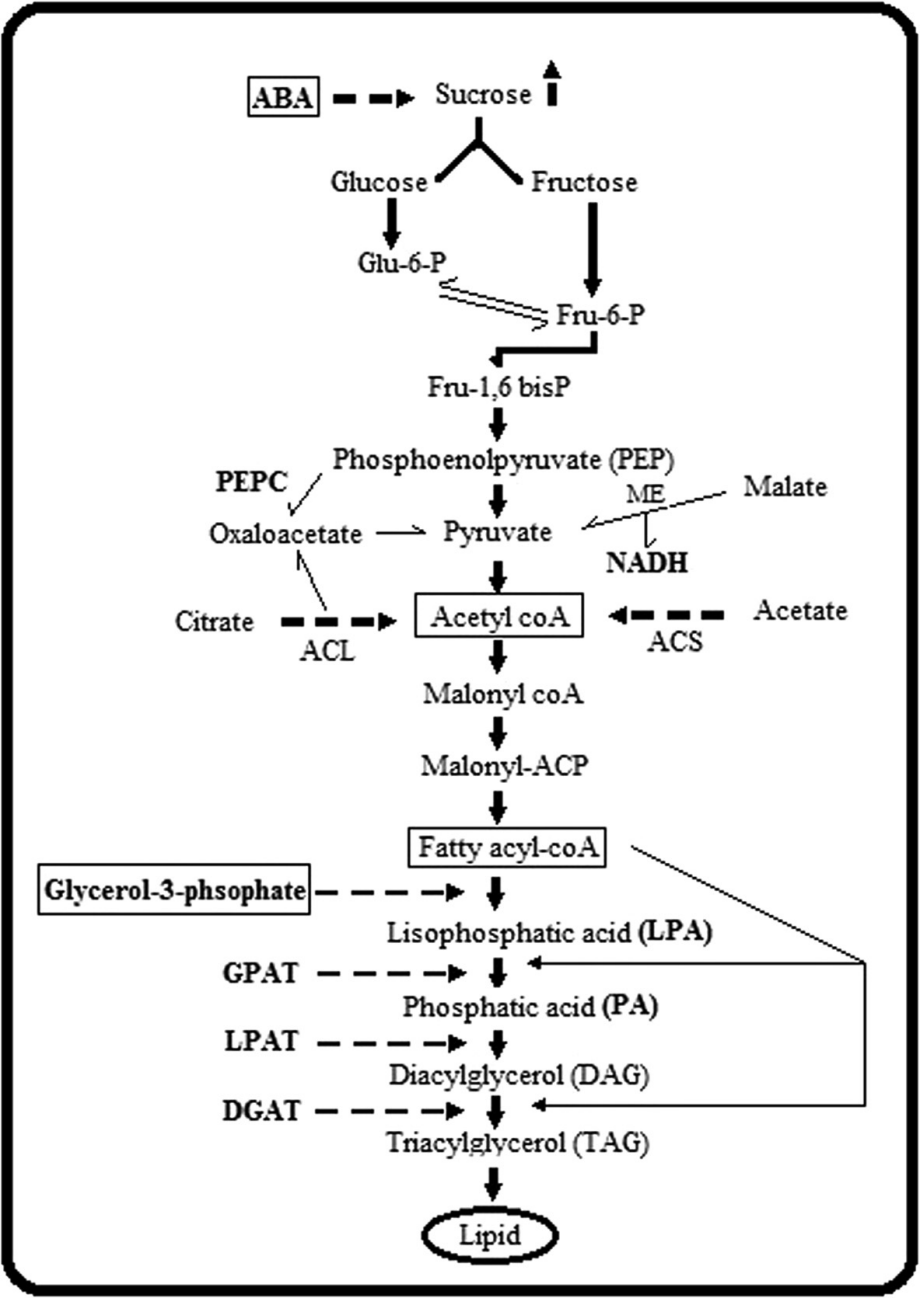
**Overall view of ABA signaling towards sucrose metabolism and lipid biosynthesis in castor developing seeds.** This pathway describes major genes participating in sucrose and lipid metabolic pathways like Glucose-6-phopshate, fructose 1,6 bisphosphate, pyruvate kinase, glycerol-3-phosphate, ACS, GPAT, DGAT positively regulated to exogenous ABA signaling (Additional file 4: Table S1).

In the total DEGs identified, several genes were found to be directly or indirectly involved in critical plant metabolic pathways related to seed filling like sucrose, starch, cellulose, lipid and protein metabolism apart from genes participating in other regulatory pathways. As shown in Additional file 4: Table S1, crucial enzymes like glucose 6 phosphate [30170.m014025], fructose 6 phosphate [30189.m001668], fructose 1,6-bisphosphate [29576.m000229], glycerol-3-phsophate [30131.m007256, 30147.m014220], 3- phosphoglycerate dehydrogenase [30074.m001363], polyethanolpyruvate [PEP] carboxynase [30170.m013617], pyruvate kinase [29815.m000503, 28842.m000927], pyruvate dioxygense [29993.m001037], pyruvate decarboxylase [29601.m000442, 29900.m001589], NAD(P)H dehydrogenase [29933.m001426], NADH dehydrogenase [53582.m000022], NADH oxidoreductase [48739.m000082], malate dehydrogenase [30138.m004069], glutamate decarboxylase [29780.m001375], acetyl CoA dehydrogense [30111.m000733], 3-OH acetyl CoA dehydrogenase [29912.m005496], acetyl CoA transferase [29844.m003188] participating in sucrose metabolic pathway i.e., breakdown of sucrose for synthesis of storage reserves (Figure 4). Sucrose synthase 3 (SS) [29986.m001646] a key enzyme in the conversion of sucrose in to starch was highly regulated by exogenous ABA signaling. In support, critical enzymes participating in cellulose synthesis like cellulose synthase [30073.m002256, 29603.m000538], UDP glucosyltransferase [29724.m000846, 29678.m000511, 30138.m003909, 30138.m003910], glucan endo 1,3 beta glucosidase [30170.m014027], glycosyl transferase [30138.m003935], xyloglucan [30179.m000573], beta 1,3 glucuronyltransferase [30170.m013621] were highly expressed. In addition, eight sugar transporter genes (STP) which play a nutritional role in supplying sugars to the cells for growth and development [29912.m005520, 30147.m014261, 29726.m003958, 29667.m000356, 29693.m001965, 29844.m003367, 29844.m003369, 29933.m001432] were found to be highly responsive supporting the high uptake of total sugars mediated by exogenous ABA signaling.

Contrarily, some crucial genes participating in the sucrose metabolic process were also down-regulated. These include pyruvate dehydrogenase [28883.m000717], 3-phopshoglycerate dehydrogenase [3-PGA] [29801.m003106], NADP malic enzyme [29794.m003406], NADH dehyrogenase [29726.m003961], UDP-glucosyltransferase [29970.m000993], cellulose synthase [29863.m001055], UDP glucose 6-dehydrogenase [30174.m009132] and PEP carboxynase (ATP) [28179.m000470]. As a result of the mediation of crucial sucrose metabolic enzymes; rate limiting enzymes participating in lipid biosynthesis as well as metabolic pathway (Figure 4) were highly triggered. Of the positive responsive transcripts due to the effect of exogenous ABA, several rate limiting enzymes involved in acyl CoA dependent lipid synthesis pathway like acyl coA synthase (ACS) [29844.m003365], Acyl coA binding protein (ACBP) [30170.m013760], type 1 diacylglycerol acyl transferase (DGAT1) [29912.m005373], ER glycerol-3-phosphate acyltransferase (GPAT 2 and 3) [30174.m008615, 29969.m000267], and desaturase genes participating in acyl coA independent lipid synthesis pathway like acyl carrier protein desaturase (SAD1) [29929.m004514], omega-6-desaturase (FAD2) [29696.m000105], fatty acid desaturase (FAD3) [30174.m008679] genes were observed to be highly modulated with a high fold change (≥1) in castor bean developing seeds at this stage (Additional file 4: Table S1). Lipid breakdown genes like long chain fatty acid ligase (LCFA) [30076.m004616], phospholipase D (PL) [29784.m000369], monoglyceride lipase (MAGL) [29986.m001619], diacylglycerol synthase (DAGS) [28726.m000069] and triacylglycerol lipase (TAGL) [30193.m000713, 29158.m000191, 29726.m004101, 29904.m003044, 29620.m000558] were also influenced by ABA signaling. Some significant enzymes related to fatty acid β-oxidation like 3 ketoacyl CoA thiolase B (KAT2) [29904.m002984], 3-OH hydroxy acyl CoA dehydrogenease (29668.m000313) were also triggered by exogenous ABA activity (Additional file 4: Table S1).

In addition, cytochrome b5 [30213.m000673] a rate limiting enzyme (electron supply) for the synthesis of ricinoleic acid in castor bean seeds showed high expression comparing to control levels (Additional file 4: Table S1). On the other hand, critical fatty acid synthesis genes like ketoacyl synthase (KAS) [29693.m002034], keto acyl reductase (KAR) [29929.m004732], biotin carboxylase subunit of Het-ACCase (BACC, BCCP) [30185.m000954, 29630.m000809] and fatty acid desaturase [29794.m003308] were found to be down regulated by the exogenous ABA signaling (Additional file 4: Table S1). Although the total protein content was slightly decreased (Table 1), we examined the gene models encoding protein showing differential response for exogenous ABA as oil (45%) and protein (34%) comprise the major proportion of the storage reserve complex in castor bean seeds. 7S globulin [29993.m001039, 29844.m00331], cytochrome P450 [29970.m001003 (CYP81D1), 30170.m013965 (CYP2), 30170.m013873 (CYP707A4), 29813.m001518 (CYP72B1), 30115.m001196 (CYP707A4), 30174.m008617 (CYP734A1), 30076.m004534 (CYP72B1)], late embryogenesis abundant (LEA5) [29601.m000448, 29726.m004064], early nodulin [29983.m003167], zinc finger protein [28152.m000908, 28637.m000203, 29589.m001255, 30170.m013751, 29589.m001278], RING H2 finger protein [29810.m000139, 29986.m001641, 29048.m000065, 29983.m003157, 30078.m002255] and SGP1 G-protein [29666.m001485], were among the protein encoding genes playing vital role in seed development positively regulated with fold change ≥1 (Additional file 4: Table S1). Several other critical genes encoding storage and developmental proteins were down regulated under ABA signaling (Additional file 4: Table S1). These include cytochrome P450 [30094.m000683 (CYP711A1), 30128.m008568 (CYP51)], ring H2 finger protein [30068.m002520, 29603.m000511], glycine rich cell wall protein [30010.m000662], proline rich cell wall protein [29724.m000836] and cell division control protein [30171.m000404].

The intensity of ABA signaling pathway is well known to be controlled by multiple factors. Several physiological responses of seeds are closely regulated by the network of transcription factors (TFs). In relation to this, several seed specific transcription factors analyzed showed differential response for exogenous ABA. Among such MYB factor [30190.m011160, 30055.m001589], r2r3 MYB factor [30066.m000714; 28154.m000040; 29686.m000890; 30170.m013698], TINY [29938.m000611], F-box and wd 40 domain protein factor [29851.m002476], CCAAT binding factor (NF-YB6) [29656.m000483; 29912.m005288], WRKY factor [30147.m014474, 30174.m008663], binding factors related to ABI3/VP1 (RAV1) [29738.m001050], long chain fatty acid cofactor [27446.m000490], NAC domain factor [30076.m004490, 30076.m004486], bZIP [29634.m002076, 29646.m001108, 29832.m000310, 30076.m004659, 30131.m007163, 30170.m013868] and Bhlh factor [29827.m002528] showed up-regulated expression (Additional file 4: Table S1). These TFs identified belong to several families including bZIP, CCAAT, PHD, ARF, bHLH, NAC, MYB, WRKY and AP2/EREBP and of these families, number of TFs (homologous) identified have been previously reported to regulate seed development [15] (Additional file 4: Table S1). Very few transcription factors belonging to CCAAT, NAC domain, MYB, PHD family were down regulated by the influence of exogenous ABA (Additional file 4: Table S1). As shown in Additional file 4: Table S1, some of the genes directly participating in pentose phosphate pathway were also strongly influenced by exogenous ABA. Genes like ribose 5 phosphate isomerase [29932.m000590], Ribose-phosphate pyrophosphokinase [30054.m000794], 1-deoxyxylulose 5 phosphate synthase [29726.m003963], trehalose 6 phosphate [28863.m000088, 30147.m013978], which mediate support to sucrose assimilation process in plants oil seeds were also responsive to exogenous ABA signaling. The response of positively modulated metabolic genes were in high ratio as of lipid genes when compared to negatively regulated genes (Figure 1).

### Hormone metabolism is triggered by exogenous ABA signaling

Cross talk between ABA and other hormone signaling pathways were analysed, in particular genes participating in gibberellin signaling, ethylene-mediated signaling, auxin-mediated signaling to exogenous ABA signal. Genes like gibberallin receptor GID1 [30170.m013907], indole 3 acetic acid synthase GH3.6 [27533.m000079], chitin inducible giberallin responsive protein [29648.m001919], ethylene receptor [29603.m000534], brassinosteroid regulated protein BRU1 [30065.m001165], auxin induced protein 5NG4 [29783.m000310], auxin responsive protein IAA1 [30169.m006326, 29841.m002749], auxin repressed protein [30190.m011325], auxin carrier protein [29816.m000677], ethylene Insensitive 3 [29708.m000185] showed high induction fold towards exogenous ABA (Additional file 4: Table S1). In addition, 9-cis-epoxycarotenoid dioxygenase (NCED1, 3) [30202.m000262, 29693.m001992] gene along with beta carotene hydrolase [30169.m006624] which function as key enzyme in ABA biosynthesis were highly expressed indicating the up regulation of ABA levels in the castor developing seeds as a result of exogenous ABA activity (Additional file 4: Table S1). NCED1 was the predominant ABA biosynthesis gene with an induction fold of 8.11 towards exogenous ABA signaling. Many protein phosphatase 2C [29794.m003349, 29912.m005442, 30169.m006515, 28211.m000131, 30189.m001658] genes which are negative regulators (i.e., low expression levels in seed) for ABA in seed were highly up-regulated. Contrarily, several genes related to hormone signaling involved in seed development like gibberallin 2 oxidase [29851.m002508], Indole 3 acetic acid ARG7 [30131.m007151], Brassinosteroid receptor kinase [27985.m000860], Brassinosteroid insensitive 1 [29631.m001053], Chitin inducible giberralin protein [29949.m000124] were down regulated by ABA signaling (Additional file 4: Table S1).

### sqRT-PCR analysis confirms DGE validation

**Figure 5:**
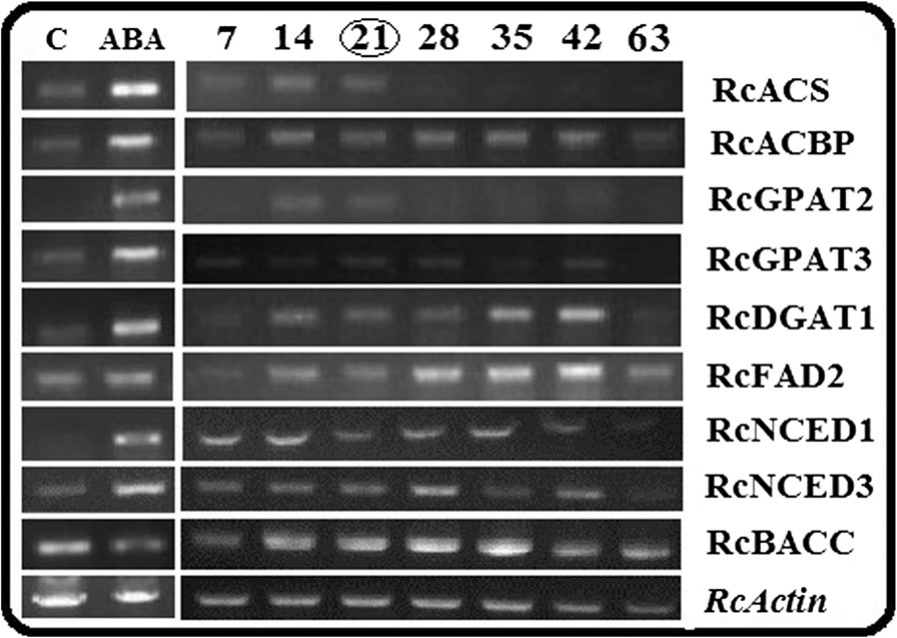
**RT-sqPCR validation of differentially expressed genes (DGEs) identified from high throughput RNA-sequencing analysis [left].** Total RNA used for RNA-Seq (21 DAP) was used for sqRT-PCR. Expression profiles during different developmental stages (7-63 DAP) [right]. The PCR primers are shown in Additional file 5: Table S2.

In order to confirm the ABA regulatory changes validated by DGE analysis, *sq*RT-PCR analysis was performed with lipid and ABA biosynthesis genes. We randomly selected seven lipid genes encoding the key enzymes in lipid biosynthesis pathway which were differentially expressed along with two ABA biosynthesis genes. Acyl coA synthase (*RcACS*), Acyl coA binding protein (*RcACBP*), ER glycerol-3-phophate acyltransferase (*RcGPAT2* and *RcGPAT3*), type 1 Diacylglycerol acyltransferase (*RcDGAT1*), Omega-6 desaturase (*RcFAD2*) and the two ABA biosynthesis genes (*RcNCED1* and *RcNCED3*) showed positive response to ABA treatment with a negative response being observed for biotin carboxylase subunit of Het-Accase (*RcBACC*) gene (Figure 5). In order to broaden our knowledge on expression profile ration of these genes after ABA treatment, we also studied the expression patterns at different seed developmental stages (7-63 DAP) (Figure 5). From our results, genes like *RcDGAT*, *RcFAD2*, *RcNCED1* and *RcNCED3* were expressed at all the stages with exceptions of genes like *RcGPAT2* expressed in early to mid developmental stages. From these results it was apparent that *sq*RT-PCR results were consistent with the DGE data and showed up regulation of all the six lipid genes that were up regulated in DGE analysis (Figure 5).

## Discussion

Seed filling, a phase defined by morphological, cellular, and metabolic changes in the endosperm and embryo that coincide with rapidly increasing storage reserves, determine seed development and storage reserve accumulation [25]. Although diverse functioning ABA responsive factors were identified in plants, relatively limited information is known on ABA responsive genes involved in storage reserve accumulation during seed filling. Thus, the present study is focused on analyzing the effect of exogenous ABA signaling on storage reserve accumulation during seed filling at both physiological as well as at transcriptional level.

Since most metabolic pathways of storage reserve biosynthesis are branched, and there are alternation routes, futile cycles, and product turnover in developing seeds, the molecular mechanisms determining the differential partitioning of seed reserves into the major storage components remain largely unknown [26]. Lipid and protein are the major storage reserves in castor bean seeds [18]. Earlier studies reported that exogenous ABA promoted lipid accumulation in developing seeds of *Picea glauca*[10], developing wheat embryos [27] and embryo cultures of rapeseed [7] under *in vitro* conditions. In support to this, our current study demonstrated that exogenous ABA enhanced the sugar accumulation (6.3% increases) followed by a significant storage lipid enhancement (4.9% increases) in castor developing seeds. Triacylglycerols (TAGs) are the major storage lipids in diverse oilseeds [28]. Storage TAGs in the developing seeds of castor bean are highly enriched with unusual fatty acids comparing to phospholipids [29]. Thus our study particularly focused on neutral lipids containing different class distribution of TAGs (Additional file 1: Figure S1). As a highly reduced form of carbon, TAGs serve as an important energy reserve in plant seeds, providing for subsequent germination and seedling development. Marked effect of exogenous ABA towards TAG lipid classes, in particular TR2 and TR3 denoted the inductive action favoring unusual fatty acid synthesis, similar to the effect observed in isolated developing wheat embryos [27]. A major metabolic activity in developing oilseeds is the conversion of sucrose via glycolysis to lipids [30], indicating that exogenous ABA might have resulted in an increased conversion of sucrose via glycolysis to lipids in the cultured seeds. Previous observations found that exogenous ABA enhanced the accumulation of total sugars in cultured seeds of soybean and *Arabidopsis* because of the high uptake of sucrose from nutrient media as a result of alteration in the osmotic potential caused by exogenous ABA signals [31-33]. In support to this, sugar levels in particular were measured in the present study as they are the major transport carbohydrate in oilseeds and also work as an important signaling molecule that regulates genes involved in photosynthesis, metabolism, and developmental processes [34]. During seed germination and seedling development, storage TAGs are cleaved by lipases from their glycerol backbone in the oil body, and convert to carbohydrates major as sucrose for transport to the root and shoot axes [35]. From the prolific comparison of metabolite levels after ABA treatment in developing seeds; it is noteworthy to mention that high uptake of sucrose (after ABA treatment) from the culture medium as well as the high proportion of sucrose synthesis (*in vivo* at 21 DAP) at this stage of development might have added to the pushing of carbon fluxes towards TAG accumulation along with a strong interaction between ABA and other carbohydrate metabolites (Additional file 2: Figure S2). This is in agreement with Schussler et al. [31] who elaborated the ABA stimulation on high uptake of sucrose in excised soybean embryos cultured under *in vitro* conditions [31]. Based on our results and other reports it seems reasonable to think that the substantial increase in carbon assimilation in the form of sugars towards the TAG accumulation mediated by exogenous ABA might be the possible reason for the significant increase in lipid deposition without causing much difference in the dry weight proportion in developing castor seeds.

The *de novo* synthesis of fatty acids in plants occurs in the plastids through the activity of fatty acid synthetase. The synthesis of the malonyl-coA enzyme that is required for acyl-chain elongation requires the import of metabolites (participating in the sucrose breakdown) from the cytosol and their subsequent metabolism. The biosynthesis of lipids in plant seeds is dependent on the supply of many cytosolic metabolites, such as sucrose, acetate, malic enzymes, glucose 6-phosphate, pyruvate, glycerol 3-phosphate [36]. In the current study, enhancement of sugar sink strength may directly push the carbon fluxes toward lipid biosynthesis, or indirectly enhance sugar metabolism, and correspondingly provide much more precursors for seed lipid biosynthesis. In addition, our current study showed that exogenous ABA application slightly decreased the protein content in cultured castor seeds. Exogenous ABA transiently decreased the protein content at the early stages (21 DPA) of grain filling period in wheat [37]. Similar effect of exogenous ABA on enhancing lipid deposition with decrease in protein content has been previously reported in the embryos of *Brassica napus* L [7]. Decrease in the enzyme activity of ammonia assimilation and substrate concentration of protein synthesis due to exogenous ABA action might have caused the reduction in protein concentration in castor developing seeds in agreement with the previous observation during wheat grain filling [37]. In addition, ABA induced a decline in total nitrogen fixation resulting in a decrease in protein content after 5d of treatment independent of sucrose synthase (SS) activity (enzymes participating in starch synthesis) in seeds of pea [38]. These clearly state that exogenous ABA induces a switch of the conversion from carbon fluxes toward lipids and proteins. Contrarily, some studies showed that the exogenous ABA enhanced accumulation of seed storage proteins in soybean embryos [39], *Arabidopsis*[40] and date palm [41] independent of other storage metabolic pathway. From our results and previous reports, it is evident that exogenous ABA effect on protein accumulation in *in vitro* cultured seed is stage specific and species dependent.

Through DGE analysis, we obtained a total of 2568 differentially expressed transcripts, in which 1507 transcripts were expressed significantly higher and 1061 transcripts had a down regulated expression in ABA treated library (Figure 1). This discrepancy is a reflection of the effective signaling of ABA in to the seed tissues, in which genes associated with seed metabolic pathways are likely highly regulated. Among these differentially expressed transcripts, more number of cytosolic metabolites participating in sucrose breakdown was highly expressed in ABA treated seeds, respectively (Additional file 4: Table S1). Positive utilization of sucrose metabolites like NADPH, malic enzyme (ME), Glutamate-to-γ-Aminobutyrate to improve storage reserves have been reported in both plant and microbial systems [42]. Analysis on differential expression of genes encoding enzymes involved in fatty acid and TAG biosynthesis may provide clues to identify the key genes that play pivotal roles in the limiting steps of storage lipid biosynthesis which is consistent with the previous observation of ABA influence towards triglyceride content and lipid biosynthesis genes in seed cultures of *Brassica napus*[7,43]. Several rate limiting transcripts related to lipid biosynthesis and metabolism were found at higher expression levels in ABA treated seeds (Additional file 5: Table S2). Associated with lipid synthesis and metabolism, transcripts of acyl coA synthase (RcACS), diacylglycerol acyltrasnferase (RcDGAT), ER glycerol-3-phosphate (RcGPAT), fatty acid desaturase (RcFAD), acyl carrier desaturase (RcSAD), triacylglycerol lipase and long-chain-fatty-acid-CoA ligase (LCFA) were shown significantly higher expression levels After ABA treatment. Furthermore, six highly expressed bZIP transcription factors identified may be involved in regulating storage reservoir accumulation during seed filling in castor bean. In addition, differentially expressed transcripts were significantly enriched in seven GO terms, suggesting that ABA signaling may be complex and participate in diverse biological processes during seed filling.

The sqRT-PCR technique provided a sensitive and specific method to analyze multigenic expressions, enabling quantification of the weakly expressed transcripts in the picogram range of RNA allowing diverse studies of gene expression [44]. Fully replicated sqRT-PCR analysis, to certain extent, complements the lack of DGE replications. All eight lipid genes subjected to the sqRT-PCR analysis in this study showed the similar trends with the DGE results, suggesting that our results be confirmed.

## Conclusion

In conclusion, the present study showed that the application of exogenous ABA enhanced the storage reserve accumulation together with the global regulatory changes at transcriptional level towards seed filling. These findings provide a comprehensive coverage to discover many known genes of several major metabolic pathways substantially responsive to exogenous ABA signaling, which also serve as a potential contribution to available sequence resources for castor bean. From our results and diverse assessment, it was conclusive that ABA exhibits a stage specific and species dependent functioning when applied as an external supplement during seed filling process under *in vitro* conditions. These results serve as basic information to dissect the physiological mechanisms underlying ABA signaling towards seed filling and storage reserve accumulation in castor bean seeds.

## Materials and methods

Seeds of castor bean var. ZB306 (kindly provided by Zibo Academy of Agricultural Sciences, Shandong, China) were germinated in Xishuangbanna Tropical Botanical Garden (21°560 N, 101°150 E, 600 m asl) under natural conditions. Mature female flowers were hand pollinated and tagged as 0 days after pollination (DAP). Developing seeds with capsulate at ca. 21 DAP were collected from the field. Young seeds were separated from capsulates and were stored at room temperature (18-25°C) for several hours. Decapsulated seeds were inoculated in MS media (Murashige and Skoog) supplemented with exogenous ABA (Sigma, China), as described previously [24], at 1, 10, 50 and 100 μM concentrations to sort out the optimal ABA concentration for further test. Same treatments without ABA in MS media were used as control. Once the optimal ABA concentration was determined, two decapsulated seeds from the same capsulate were considered as a pair for *in vitro* seed culture, one was cultured with ABA in MS media, and other as control (without ABA). At least five individual experiments were replicated. Tested seeds were dried overnight in Eppendorf tubes under vacuum centrifugation. The dried seeds were weighted (DW, dry weight). The contents of storage materials including total lipids, proteins and total sugars were measured for each cultured seed after 96 h incubation in dark at 25-28°C as well as in developing seeds. For the preparation of RNA samples, three seeds cultured with ABA for 24 h and the other three seeds from same capsulate cultured without ABA were collected as tissues for extracting RNA.

### Measurement of storage materials

For lipid extraction, five replicate samples of cultured seeds (for cultured ABA seeds or controls, respectively) were weighted and frozen in liquid nitrogen for a few minutes, and ground to fine powder in a mortar. The fine powder was transferred into 10 ml glass tubes, and the total lipid was extracted using hexane–isopropanol method described previously [45]. For total protein measurement, as described by Borek et al. [26], five replicate samples of cultured seeds for ABA or controls were weighed and homogenized (Fluko, China) using 0.1 M TRIS-HCl buffer, pH 7.8. The homogenate was centrifuged for 20 min at 22 000 *g*[26]. Protein concentration in the supernatant was determined according to [46], using bovine serum albumin as a standard [46]. For sugar estimation, a previously described dinitrosalicylic acid (DNS) colorimetric method was applied with minor modification [47]. Briefly, seeds of 0.5 g in weight were homogenized and extracted twice with 90% ethanol. The supernatant (ethanol extracts) were collected and pooled to a final volume of 10 ml with double distilled water. A suitable aliquot (1 ml) was taken from the extract and 20 μl of concentrated hydrochloric acid was added and allowed to hydrolysis for 5 min at 90°C after which 0.05 ml of 5N KOH was added to neutralize the acid (alkaline condition for color development). Finally, 0.5 ml of DNS solution (0.1%) was added to the solution and kept for 10 min at 95°C boiling water. The solution was then cooled for color development and the absorbance of this solution was measured at 540 nm using a UV-VIS spectrophotometer (Model DU 640B, Beckman, USA). Student’s *t*-test was statistically used for all significance tests.

### High-throughput RNA-tag sequencing and analyses

The total RNA was isolated from three replicated seeds cultured with ABA for 24 h and three control samples, respectively. RNA extraction was performed according to the manufacturer’s instructions of Trizol reagent (Invitrogen, USA) and purified using an RNeasy Mini Kit (Qiaden, Hilden, Germany). The total RNA was checked for quality and quantity using a Nano-Drop ND-1000 spectrophotometer, and the high quality RNA was used to construct tag libraries respectively for deep-sequencing. Briefly, total RNA (about 6 μg) was enriched by oligo (dT) magnetic beads and oligo (dT) beads were used as primer to synthesize the first and second strand cDNA. The cDNA was digested by two types of endonuclease: Nla III or Dpn II, acquiring 17 bp tags with different adaptors of both ends to form a tag library. After 15 cycles of linear PCR amplification, 105 bp fragments were purified by 6% PAGE gel electrophoresis. After denaturation, the single-chain molecules were fixed on to the Illumina Sequencing Chip. Each molecule was then allowed to grow into a single-molecule cluster sequencing template through *in situ* amplification. Four colored nucleotides were added for sequencing using the method of sequencing by synthesis (SBS). Millions of raw reads were generated with a sequencing length of 49 bp. Sequencing was performed in BGI Shenzhen (China). The raw data from the ABA treatment and control tag libraries of castor bean were preprocessed to filter out low quality reads and clipped adapter sequences. After that, all clean reads were mapped to the castor bean genome (http://castorbean.jcvi.org/index.php) to obtain unique reads and reads abundance using SOAP2 software [48]. To compare the differential expression of genes between ABA treatment and control libraries, the expression level of each gene in the libraries was normalized to the number of transcripts per million (TPM). Genes with significantly different expression were determined by P < 0.05 and fold-change ≥ (log2 (sample1/sample2)) in two samples. To dissect the potential functions of genes, GO enrichment and KEGG pathway analyses were performed. For enrichment analysis of the function and pathway, we mapped all differentially expressed genes to terms in GO and KEGG database.

### *Semi-quantitative* RT-PCR analysis

Total RNA from the same samples used for high-throughput Tag-Seq sequencing was used for sqRT-PCR experiments. A first-strand cDNA fragment was synthesized from total RNA using Superscript II reverse transcriptase (Invitrogen, USA). Gene-specific primers were designed according to the gene sequences using the Primer Premier 5.0 (Premier Biosoft International, Palo Alto, CA). RT-PCR amplifications were performed in a *GeneAmp*® *PCR System* (Applied Biosystems). The optimized PCR reactions (25 μl) contained 200 ng of first-strand cDNA template, 2.5 μl 10 × PCR buffer, 0.5 μM of each primer, 0.25 mM of dNTPs, and 1.2 μM of Trans-start™ HiFi DNA polymerase (Transgen, Beijing, China). The following PCR conditions were used: initial denaturation at 95°C for 1 min, 30 cycles of denaturation at 94°C for 30 sec, annealing at 55-58°C for 20 sec and extension at 72°C for 5 min. The castor bean *Actin* gene was used as internal control to normalize the relative amount of mRNAs for all samples. The amplified products were analyzed by electrophoresis system in 1% agarose gels and visualized by ethidium bromide staining. The primers used in this study are listed in Additional file 5: Table S2.

### Extraction and quantitation of endogenous ABA levels in treated seeds and culture medium

Pre-weighed seed homogenates were prepared from harvested seeds as described by Holbrook et al. [49] and adjusted to give an FW equivalent of 250 mg FW/mL homogenate. Homogenates equivalent to 1g FW were lyophilized for 5 to 8 h 1% butylated hydroxytoluen (BHT). The residue was stirred at room temperature overnight, centrifuged at 2500 *g* for 20 min to pellet undissolved solids, and the supernatant, containing the free ABA and metabolites, was collected. In addition, Samples of culture media (24 h treated) were filtered through 2-μm nylon filters directly into sample vials [7]. Because of the liability of the free ABA, samples were analyzed immediately or frozen at -80°C overnight and analyzed using a simplified ELISA technique (free of internal matrix) [50].

## Competing interests

The authors declare that they have no any competing interests.

## Authors’ contributions

UC designed and carried out all the experiments and also wrote the manuscript. WX helped in providing the seed samples. AL is the creative head for this research. All authors read and approved the final manuscript.

## Supplementary Material

Additional file 1: Figure S1 Lipid class distribution of neutral lipids separated by Thin Layer Chromatography plate (TLC) (i) TR1- triglycerides of two or three unhydroxylated acyl moieties (ii) TR2- triglycerides containing two ricinoleates and one unhydroxylated acyl moiety (iii) TR3-triricinoleins (control (C), ABA treated [left] and at different developmental stages [right]. Click here for file

Additional file 2: Figure S2 (A) Fresh weight/ Dry weight of developing seeds (7-63 DAP) (B) Lipid, protein and sugar metabolite levels at different seed developmental stages (7-63 DAP). Click here for file

Additional file 3: Figure S3 **(A)** Measured ABA levels in cultured seeds and (B) time course study of ABA levels in culture medium. Click here for file

Additional file 4: Table S1 Overall differentially expressed genes identified with ABA treatment (2568 DEGs). Click here for file

Additional file 5: Table S2 Primers used in this study. Click here for file
