# Supplementary figures and images for: Transcriptome profiling identifies ABA mediated regulatory changes towards storage filling in developing seeds of castor bean (Ricinus communis L.)
Source: Cell Biosci. 2014 Jun 30;4:33. doi: 10.1186/2045-3701-4-33 (PMC4109380; doi:10.1186/2045-3701-4-33)

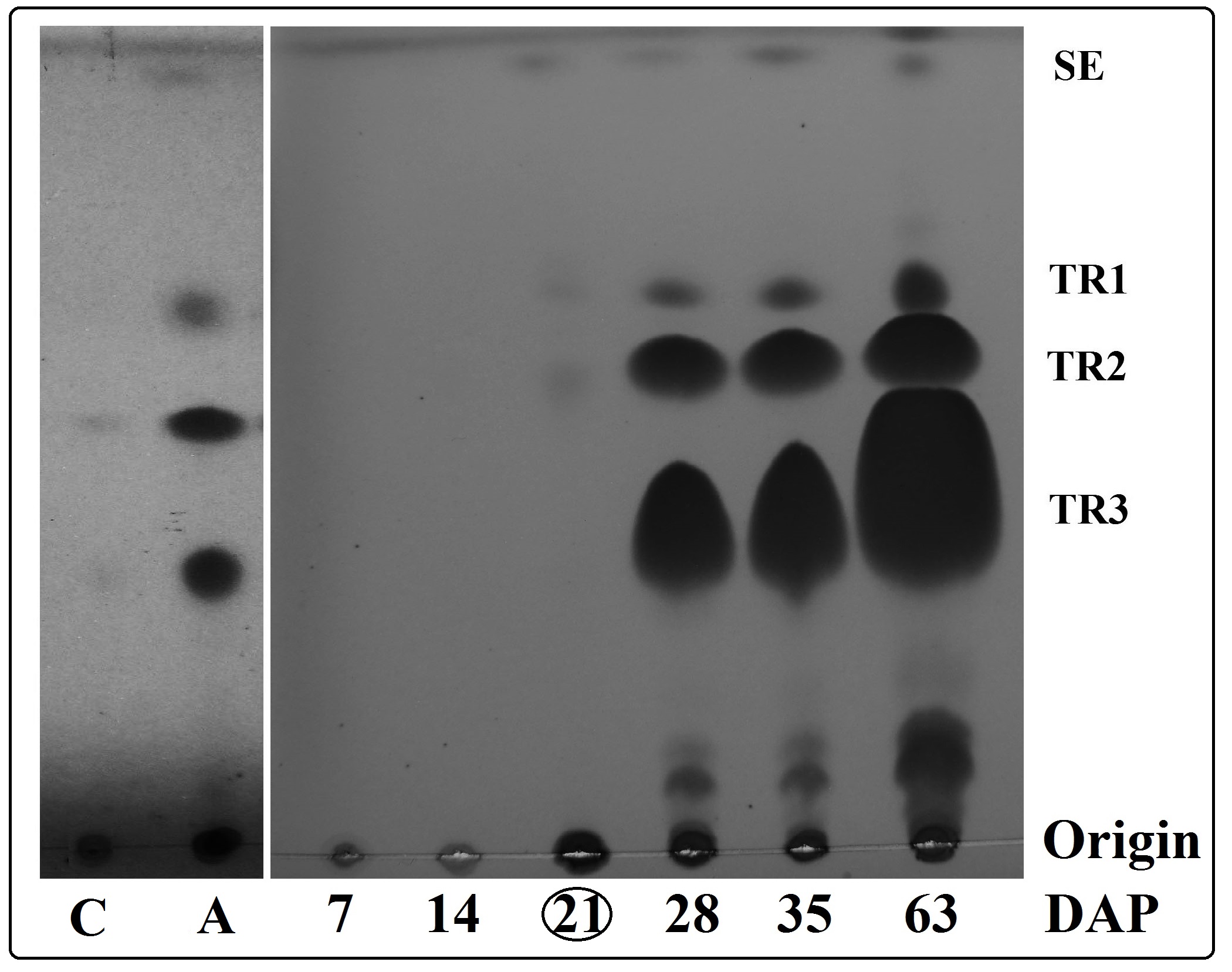

Supplement: Additional file 1: Figure S1 — Lipid class distribution of neutral lipids separated by Thin Layer Chromatography plate (TLC) (i) TR1- triglycerides of two or three unhydroxylated acyl moieties (ii) TR2- triglycerides containing two ricinoleates and one unhydroxylated acyl moiety (iii) TR3-triricinoleins (control (C), ABA treated [left] and at different developmental stages [right]. [file 2045-3701-4-33-S1.jpeg]

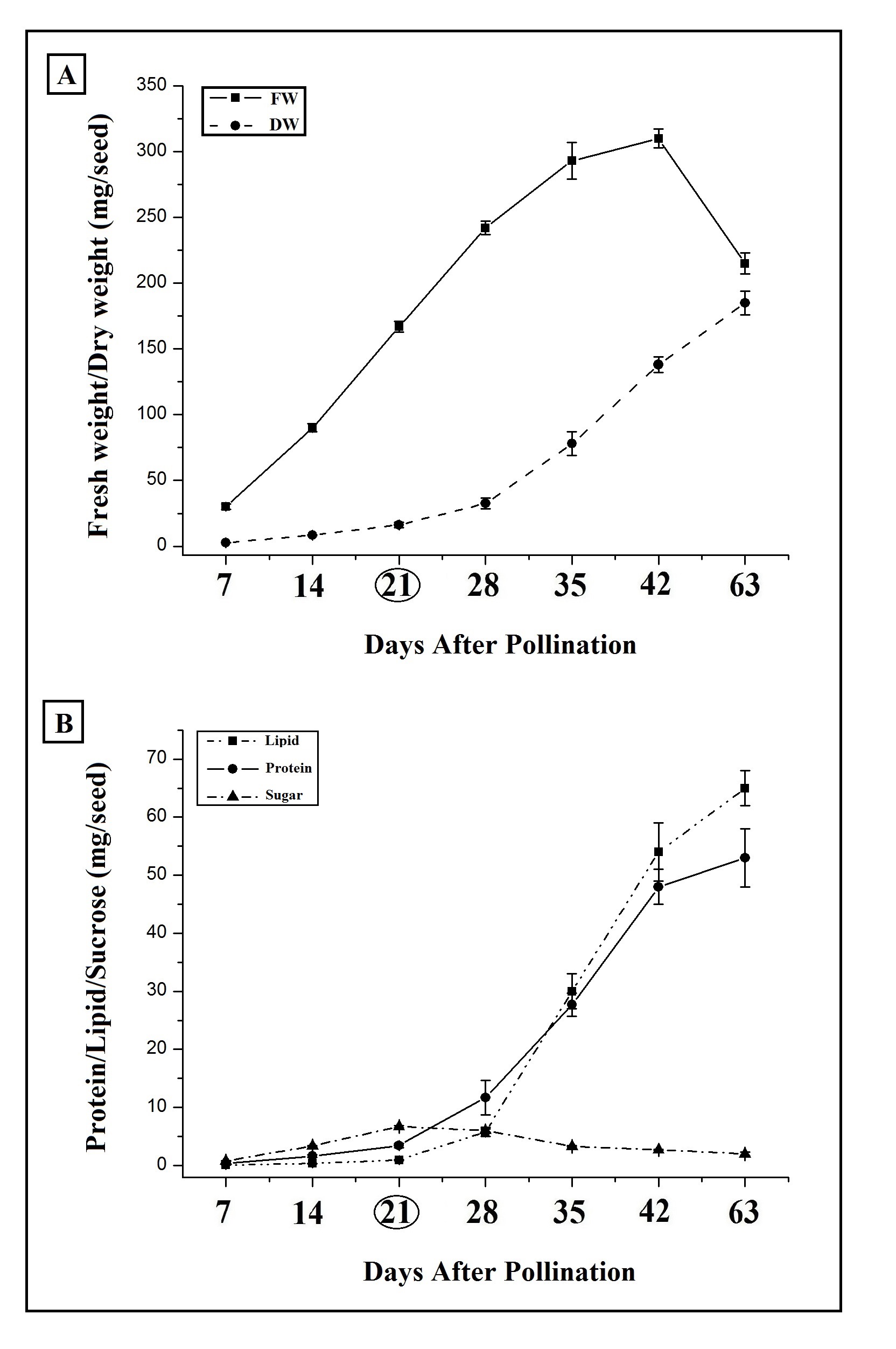

Supplement: Additional file 2: Figure S2 — (A) Fresh weight/ Dry weight of developing seeds (7-63 DAP) (B) Lipid, protein and sugar metabolite levels at different seed developmental stages (7-63 DAP). [file 2045-3701-4-33-S2.jpeg]

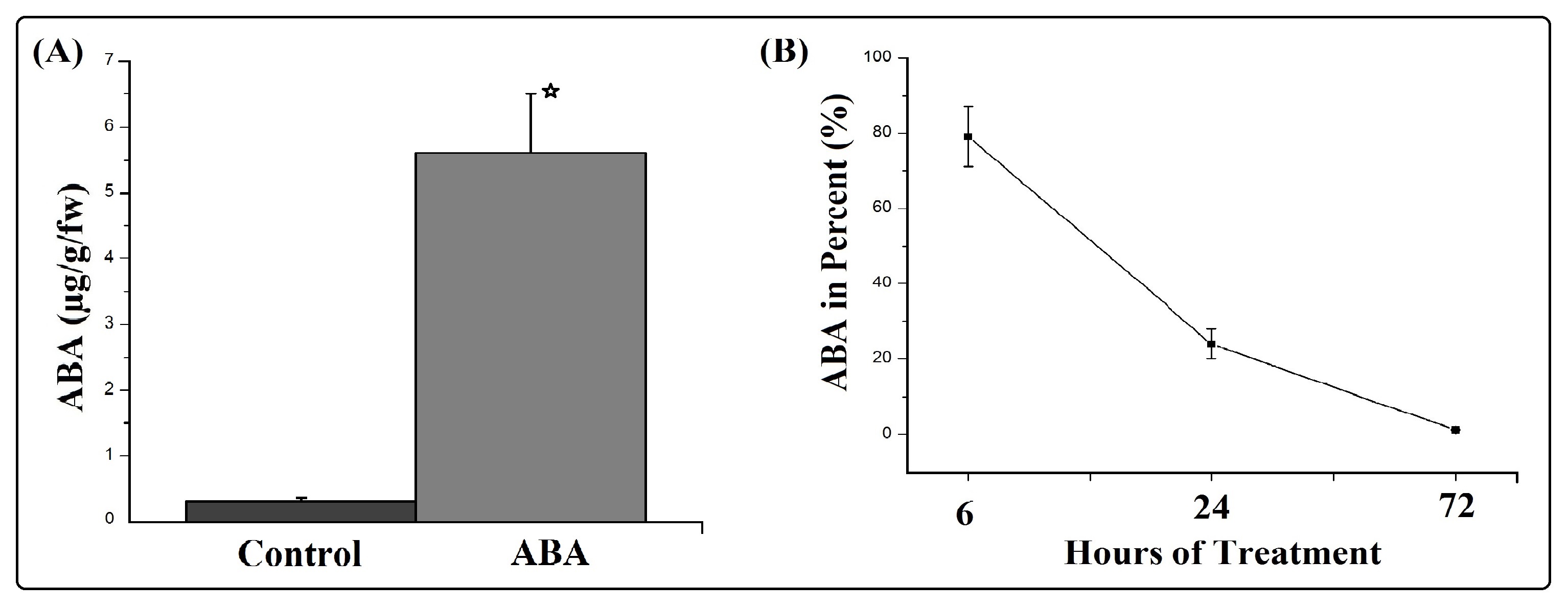

Supplement: Additional file 3: Figure S3 — (A) Measured ABA levels in cultured seeds and (B) time course study of ABA levels in culture medium. [file 2045-3701-4-33-S3.jpeg]
