# Supplementary material for: Transcriptome profiling identifies ABA mediated regulatory changes towards storage filling in developing seeds of castor bean (Ricinus communis L.)
Source: Cell Biosci. 2014 Jun 30;4:33. doi: 10.1186/2045-3701-4-33 (PMC4109380; doi:10.1186/2045-3701-4-33)
Supplement: Additional file 5: Table S2 — Primers used in this study. [file 2045-3701-4-33-S5.doc]

**Table S2** Primers used in this study for *sq*RT-PCR analysis

| **Gene name** | **Forward Primer (5’-3’)** | **Reverse Primer (5’-3’)** |
| --- | --- | --- |
| RcACS  RcACBP  RcFAD2  RcGPAT1  RcGPAT2  RcDGAT1  RcNCED1  RcNCED3  RcBACC  *RcActin* | TCCTAATCCAGCCTGCGAAA  TGGGTTTGAAGGAGGATTTTGA  GTCATGGATCTATAGGTATGTTACAGGAA  TCCATTTGCAAGGAGGGCTA  TCTATCTATCAGGTCGTGGAACGAT  GAAGCATGGCTCTGAGATTGCT  GAATTGCATGGTCACTCTGGTA  ACTCCTCCTGTTACTGACCTTCC  TCCCTCCAGGAAGCTGCA  AGTCTTGTTCCAGCCATCTCTC | ACATCTCTCGTCTGTGCTTCATCT  GCTTGCTTGTAAAGGCCATACA  AAAGCCATTTTATATCCATCAAGCA  GAGAGCATGAATCCAAATGGC  GCGGGGAATAGATCGCTTG  TTCATAAATAACTCGCCATCAGGTT  TGGTGTGTTCAAGTTGACCTTC  CCCCCTAAGATCTCCTATCTTCA  CATGTGAATAACCTCCCCACG  CAGTGATCTCCTTGCTCATACG |
